# A novel strategy for simultaneous reduction of salt and animal fat in burger using a taste contrast system based on double emulsion

**DOI:** 10.1016/j.crfs.2023.100644

**Published:** 2023-11-19

**Authors:** Hadi Hashemi, Mohammad Hadi Eskandari, Seyed Mohammad Hashem Hosseini

**Affiliations:** Department of Food Science and Technology, School of Agriculture, Shiraz University, Shiraz, Iran

**Keywords:** Double emulsion, Fat replacer, Oleogelation, Processed meat, Salt reduction, Taste contrast

## Abstract

The work investigated a taste contrast strategy to reduce the salt content in burgers by a novel design of water in gelled oil in water double emulsion (DE) as an animal fat replacer. Oleogelation reduced the particle size and improved emulsion viscosity, resulting in more emulsion stability than conventional DE. Moreover, oil gelation enhanced the encapsulation efficiency of salt. The partial substitution of the optimized DE incorporating salt within the W_1_ and cinnamaldehyde within the oil phase with animal fat in the burger successfully reduced salt content by up to 25% while maintaining the desired level of saltiness. The presence of cinnamaldehyde also increased oxidative stability and decreased color changes during storage. The replacement of DE and oleogel in burgers diminished cooking loss, while negatively affected the textural properties. Therefore, further optimization of this strategy could lead to healthier food formulations with reduced fat and salt content.

## Highlights

• A novel strategy was used to simultaneously reduce salt and animal fat in burger.
• Water in gelled oil in water double emulsion was designed as a novel taste contrast approach.
• The taste contrast strategy reduced salt content by up to 25%.
• The gelled state of oil phase enhanced the EE and perceived salt intensity.
• Water in gelled oil in water double emulsion facilitate innovative development of healthy products.

## Graphical abstract

**Figure:**
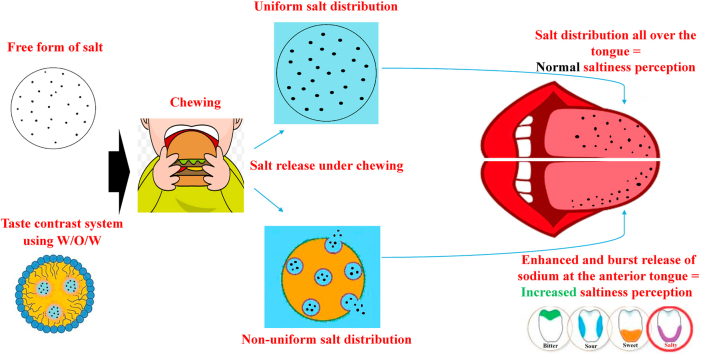

## 1 Introduction

Based on dietary recommendations, the high consumption of sodium (>2 g/day) remains an epidemic risk throughout the world (Organization, 2012). The application of salt as the main source of sodium in different food products, either in brine or powder forms, results in heart, cardiovascular, and kidney diseases (Devanthi et al., 2018). Furthermore, there has been a significant rise in obesity, chronic heart disease, and type 2 diabetes as a result of people’s dietary habits towards consuming foods high in calories and fats as well as lack of physical activity (Ghiasi and Golmakani, 2022a). Therefore, the customer demand for low-sodium and low-saturated fat foods has forced the food manufacturing industry to produce salt-reduced and fat-reduced products (Dötsch et al., 2009). Today, different strategies including gradual and stepwise salt reduction (Gaudette et al., 2019), the application of salt replacers such as calcium, lithium, magnesium, and potassium chloride salts (Grummer et al., 2013; Pietrasik and Gaudette, 2014), modulation of salt crystal size (Kasprzak et al., 2019), and inhomogeneous distribution of salt in solid-based products by forming salt layers at various contents (Mosca et al., 2013) have been suggested to decrease the salt content in food formulations. Despite these outstanding strategies, the successful application of a single strategy is still challenging mainly due to the multifunctional roles of salt in food products that increase the complexity of reducing sodium in the human diet (Gaudette et al., 2019). Therefore, the loss of product functionality, desirable taste perception, and consumer acceptability limit the application of salt reduction strategies in real food formulations. In this regard, the taste contrast approach using water-oil-water (W_1_/O/W_2_) double emulsions (DE) is a relatively novel strategy for salt reduction due to their ability to encapsulate salt for protection and controlled release during oral processing. This method can successfully reduce the salt content in emulsion-based liquid and semi-solid food formulations by more efficiently releasing salt to enhance early salty taste intensity, eliciting saltiness perception by consumers (Chiu et al., 2017). On the other hand, previous researches have shown that the incorporation of emulsion droplets can enhance the perceived salty taste intensity compared to the aqueous solution without oil droplets. This fact could be attributed to the salt concentration in the aqueous phase and the suppressed contact of salt to the buccal surface via the oil droplets (Yamamoto and Nakabayashi, 1999). Moreover, the successful application of the double emulsions as fat replacers in food products was also well documented (Eisinaite et al., 2017; Serdaroğlu et al., 2016). Although, one of the main limitations of emulsion application is the oxidation of the oil phase. To this end, natural antioxidants incorporation in the oil phase of emulsion systems is a common strategy to improve oxidation stability.

It has been reported that 20% of total salt intake is derived from processed meat products (Inguglia et al., 2017). Therefore, due to the necessity to reduce sodium in processed foods, many efforts have been conducted for salt reduction in meat industries. However, salt reduction can lead to many limitations and challenges due to its effects on the product storage, technological characteristics, and sensory quality of manufactured meat products (Rysová and Šmídová, 2021). Indeed, salt is considered a multifunctional and principal component in the formulation of processed meat due to its effectiveness in developing a salty taste and emphasizing other tastes and aromas, its preservative capacity by reducing water activity, and its functional roles in color enhancement and myofibrillar proteins solubilization which increase adhesion, juiciness, and cohesiveness of processed meat products (Bae et al., 2017; Desmond, 2006; Jeong, 2017; Rysová and Šmídová, 2021). In addition, fat plays a critical role in processed meat products to provide acceptable texture and flavor properties. On the other, many health organizations recommend cutting the portion size and reducing the consumption of highly processed high-fat meat products like burgers due to the recent concerns about the negative health implications of the presence of saturated animal fat, synthetic antioxidants, and high salt content (Kumar, 2021; Teixeira and Rodrigues, 2021; Tobin et al., 2012). Therefore, salt and fat reduction in processed meat products is still a challenging issue.

Best of our knowledge, the application of the taste contrast approach using water in gelled oil in water double emulsion as a potentially valuable and novel method for reducing the negative sensory and functional effects of sodium and animal fat reduction in processed meat products has not yet been investigated. Therefore, the present study investigated a novel design of DEs stabilized by an oleogelation mechanism in the oil phase to improve emulsion stability and subsequently the release behavior of salt from the internal water phase into the saliva. To this end, water in gelled oil in water DE incorporating salt (0.75%) within the W_1_ and cinnamaldehyde (1%) within the oil phase was partially (50%) replaced with animal fat in beef burgers, and the physical properties, taste intensity, and consumer liking of low-salt and low-saturated fat burger formulated by the taste contrast system were studied.

## 2 Materials and methods

### 2.1 Materials

Refined sunflower oil (Nina Co., Kerman, Iran) and the burger ingredients were purchased from a local market. Distilled monoglycerides (MG, >90%) were from ZTCC (Henan Zhengtong Food Technology Co., Ltd, Xingyang, Henan, China). Wheat starch was kindly provided from Khoosheh Fars (Shiraz, Iran). 2-Octen-1-ylsuccinic anhydride (OSA, 97%) and cinnamaldehyde (≥95%) were from Sigma-Aldrich (St. Louis, MO, USA). All dispersions were formulated with distilled water (DW).

### 2.2 Modification of wheat starch

According to previous reports, debranching followed by OSA modification results in improved emulsifying properties of starch due to the hydrolyzation of amylopectin to short linear starch molecules during enzymatical debranching as well as higher amphiphilic character as a result of incorporating a hydrophobic part to the hydrophilic starch during OSA modification (Jain et al., 2019). Therefore, for debranching, a starch suspension (30%) was prepared in acetate buffer (50 mM, pH 5) and then heated for 45 min in a water bath at 95 °C to gelatinize. After cooling to 50 °C, the suspension was treated with pullulanase (45 U/g starch dry basis) in an incubator at 50 °C for 24 h. The debranching process was performed in a boiling water bath for 30 min. Afterward, a proper volume of alcohol was added to precipitate the starch gel followed by centrifugation at 2000*g* for 15 min. The final sedimented starch gel was washed three times with distilled water. Finally, the precipitate was collected, dried at 40 °C overnight, then ground and sieved using 70-mesh size sieve (Jain et al., 2019).

Hydrophobic modification of debranched wheat starch was done by OSA treatment as described by Gahruie et al. (2020). Wheat starch (30%) was hydrated overnight in DW. After adjusting the pH to 8.0 using 0.5 M NaOH, the ethanolic solution of OSA was added gradually at a ratio of 3 % at 25 °C. The reaction proceeded for 90 min at 40 °C and a pH value of 8. The modification process stopped by decreasing the pH value of the mixture up to 6 using 0.1 M HCL followed by removing the excess amounts of OSA by washing the slurry with absolute ethanol, at least three times. Final OSA -modified starch was freeze-dried.

### 2.3 Preparation of taste contrast system using double emulsion

First, the primary W_1_/O emulsion was prepared by adding the aqueous phase to the sunflower oil phase containing polyglycerol polyricinoleate (6%) at the ratio of 20:80 at 70 °C while continuously stirring at 500 rpm for 15 min. Emulsification was performed at 15,000 rpm for 2 min and 10,000 rpm for 2 min using an Ultra Turrax homogenizer (IKA T18, Staufen, Germany). To prepare DE, the primary emulsion (40 g) was slowly added to the external aqueous phase (60 g) incorporating de-branched OSA-modified starch (4% w/w) at 40 °C followed by emulsification at 13,000 rpm for 2 min and 10,000 rpm for 2 min. Table 1 presents the composition of all prepared DEs formulations. In the formulation of both DE1 and DE5, salt (1%) was incorporated in W_2_, while in other DEs formulations, salt was encapsulated in W_1_. DE1, DE2, DE3, and DE4 were formulated with liquid sunflower oil, while the oil phase in DE5, DE6, DE7, and DE8 samples was gelled using MG to explore the changes in the salt release and saltiness under the physical state of the oil phase. For oil gelation, MG (10%) was dissolved in the oil phase at 70 °C under stirring for 10 min followed by storage at 4 °C to gel formation. Moreover, cinnamaldehyde (1%) was incorporated into the oil phase to investigate the oxidation process in samples.

**Table 1:** Different formulations of double emulsion samples.

Sample	Salt concentration and location	Oil type	W_1_		O			W_2_		
			Water (g)	Salt (g)	Oil (g)	PGPR (g)	MG (g)	Water (g)	Modified starch (g)	Salt (g)
**DE1**	1%-W_2_	Liquid	20	–	74	6	–	137.5	10	2.5
**DE2**	1%- W_1_	Liquid	17.5	2.5	74	6	–	140	10	–
**DE3**	0.75%- W_1_	Liquid	18.125	1.875	74	6	–	140	10	–
**DE4**	0.5%- W_1_	Liquid	18.75	1.25	74	6	–	140	10	–
**DE5**	1%-W_2_	Gel	20	–	66	6	8	137.5	10	2.5
**DE6**	1%- W_1_	Gel	17.5	2.5	66	6	8	140	10	–
**DE7**	0.75%- W_1_	Gel	18.125	1.875	66	6	8	140	10	–
**DE8**	0.5%- W_1_	Gel	18.75	1.25	66	6	8	140	10	–

### 2.4 Double emulsions properties

#### 2.4.1 Morphology

The DE3 and DE7 samples were selected to study their microstructure under the effect of oil gelation using an optical microscope (Olympus CX40, Olympus Optical Co., Tokyo, Japan). For this purpose, a drop of samples was located on the glass slide and then covered gently by a coverslip. The images were captured at 100× magnification.

#### 2.4.2 Encapsulation efficiency of salt

To determine salt encapsulation efficiency, the amount of free salt in the W_2_ phase of DEs was first measured. For this purpose, 20 mL of each sample was transferred into 50 mL falcons, and centrifuged at 10,000 g for 20 min to separate the external water phase. The concentration of salt in W_2_ was measured by the Volhard method. Next, to release the encapsulated salt in W_1_, the emulsion samples were diluted 10 times with hot DW and placed in a hot water bath for 15 min while stirring. This treatment resulted in emulsion destabilization due to the dilution, high temperature, and stirring which consequently released salt from the emulsion structure. After cooling the sample at 25 °C, 2 mL of potassium ferrocyanide and 2 mL of zinc acetate were added. After 30 min, the dispersions were filtered and DW was added to the filtered solution to reach 200 mL. After that, 20 mL of the solution was poured into a 250 mL Erlenmeyer flask and mixed well with 5 mL of 4 N nitric acid, 1 mL of ammonium iron (III) sulfate saturated solution, 20 mL of silver nitrate solution, and 3 mL of nitrobenzene to form a precipitate. Finally, the obtained mixture was titrated with potassium thiocyanate solution to observe a stable brick color.

#### 2.4.3 Apparent viscosity

The DE2, DE5, and DE6 samples were selected to explore the apparent viscosity under the effects of oil gelation as well as the salt incorporation, either in W_1_ or W_2_. To this end, a MCR 302 rotary rheometer (Anton Paar, Graz, Austria) equipped with a conical plate geometry (CP25-1, diameter 25 mm, gap size 0.052 mm and cone angle 1°) was used at 25 °C.

### 2.5 Burger preparation

The formulation of four different batches of beef burgers was presented in Table 2. To prepare the samples, the frozen lean beef shank and back fat were ground separately through the 8 mm plate. Afterward, salt, water, and frozen minced onions were added and mixed for 3 min. Finally, breadcrumbs and spices were incorporated and mixed well to form a homogenous mixture. The final paste was re-grounded through the 5 mm plate and then divided into 100 g samples to form burgers (10 × 1 cm^2^) using the commercial burger-maker.

**Table 2:** Different formulations of beef burgers.

Ingredients (g)	Burger Sample			
	Control with animal fat	Formulated with oleogel	Formulated with double emulsion	Industrial control sample
**Meat**	40	40	40	40
**Beef fat**	17.5	1.9	8.6	17.5
**Onion**	10	10	10	10
**Toasted flour**	8	8	8	8
**Water in gelled oil in water double emulsion containing 3% salt**	0	0	25	0
**Oleogel containing 4.5% salt**	0	16.5	0	0
**BHT**	0	0	0	0.02
**Water**	15	15	0.25	15
**Soy protein isolate**	8	8	8	8
**Salt**	1	0	0	1
**Black pepper**	0.099	0.099	0.099	0.099
**Red pepper**	0.053	0.053	0.053	0.053
**Omani lemon**	0.053	0.053	0.053	0.053

DE7 was selected as the best sample with lower salt content regarding its high freeze-thaw stability which has been reported in our previous work (Hashemi Gahruie et al., 2023), as well as its high salt encapsulation efficiency and homogenous morphology with smaller droplets, suggesting its better stability during storage and processing. However, cinnamaldehyde (1%) as a natural antioxidant was added into the oil phase of DE during preparation. Therefore, the second formulation of the burger was prepared by partial replacement (50 %) of back fat with DE7. The third batch was formulated with partial replacement (89%) of oleogel sample containing 10% MG and 4.5% salt in sunflower oil to compare its effects as an animal fat replacer compared to DE. The fourth batch was similar to the control sample but BHT antioxidant at a concentration of 200 ppm was incorporated into the mixture to present similar oxidation stability characteristics to commercial samples in the local markets. All beef burgers were wrapped with polyethylene packaging, and frozen at −18 °C to investigate their characteristics for 3 months.

### 2.6 Burger properties

#### 2.6.1 pH

pH values of raw burgers were studied according to the AOAC 1990 using a digital pH meter (Starter 3000, OHAUS, Switzerland).

#### 2.6.2 Color

The L* (lightness), a* (redness-greenness), and b* (yellowness-blueness) of raw and cooked beef burgers were calculated by Adobe Photoshop software after photographing the samples using a digital camera (14 MP, Tokyo, Japan) within the standard chamber (Badar et al., 2023).

#### 2.6.3 Texture analysis

A texture analyzer (TA-XT2, Stable Microsystems, Surry, UK) was used to evaluate the mechanical properties of cooked burgers using a double compression test as described by Ghiasi and Golmakani (2022a). Sample cuts (2 × 2 cm^2^) were compressed with a cylindrical probe to 20% of their initial heights at 2 mm/s test speed. The obtained force-time curves were analyzed to determine the hardness, cohesiveness, chewiness, and gumminess.

#### 2.6.4 Cooking loss

The changes in the weight of burgers before and after frying at 180 °C for 5 min were calculated to evaluate the cooking loss (%).

#### 2.6.5 Cooking shrinkage

The ratio between the differences in burger diameter before and after frying and the diameter of the raw burger was calculated using calipers to report cooking shrinkage (%).

#### 2.6.6 Peroxide value

Five grams of burgers were heated in 250 mL Erlenmeyer flasks with a closed cap in a water bath at 80 °C for 3 min. For dissolving the fat of burgers, 50 mL of acetic acid–chloroform solution (3:2 v/v) was added to the flask and well mixed for 3 min. Meat particles were separated from the mixture by filtration through a Whatman filter and then 0.5 mL of saturated potassium iodide solution and 30 mL of DW were added to the filtrates while stirring. After that, the mixture was titrated with a standard solution of sodium thiosulfate (0.1 N) to disappear the yellow color of the iodine. Finally, 0.5 mL of 10% sodium lauryl sulfate and 0.5 mL of starch solution as an indicator were added, and the titration proceeded to disappear the blue color. Results were reported in meq of active O_2_ per kg oil (Teye and Boamah, 2012).

#### 2.6.7 Thiobarbituric acid reactive substance

Four g of burger samples were mixed well with 20 mL of 20% trichloroacetic acid (TCA). 10 mL of DW was then added to the mixture followed by filtration through Whatman filter paper to separate meat particles. 2 mL of filtrate was mixed with an equal amount of 2-thiobarbituric acid (0.01 M) and heated at 100 °C for 30 min in a water bath (Ghiasi and Golmakani, 2022a). The absorbance of the cooled solution was recorded at 532 nm using a UV–Vis spectrophotometer.

TBA = A_532_ × 4

#### 2.6.8 Sensory evaluation

Sensory parameters of burgers were investigated by 16 semi-trained panelists aged between 25 and 35 from the Department of Food Science and Technology at Shiraz University using a 5-hedonic scale method from dislike extremely (1) to like extremely (5). Burger samples, after 1 month of frozen storage, were cooked at 180 °C for 5 min, named with three-digit codes of random letters and numbers, and then tested by panelists under standard conditions (Moghtadaei et al., 2018). Color, texture, odor, flavor, saltiness at the beginning of chewing, saltiness at the end of chewing, and general acceptability of burgers were considered. Moreover, a commercial burger sample was also evaluated by panelists along with other samples as a positive control sample.

### 2.7 Statistical analysis

For all measurements, the mean ± SD of three replications was reported. The mean results were analyzed using a one-way analysis of variance (ANOVA) at a significance level of *P* < 0.05. The significant differences were determined by Duncan’s multiple range test using SAS software (ver. 9.1, SAS Institute Inc., Cary, NC).

## 3 Results and discussion

### 3.1 Double emulsions properties

#### 3.1.1 Morphology

**Fig. 1:**
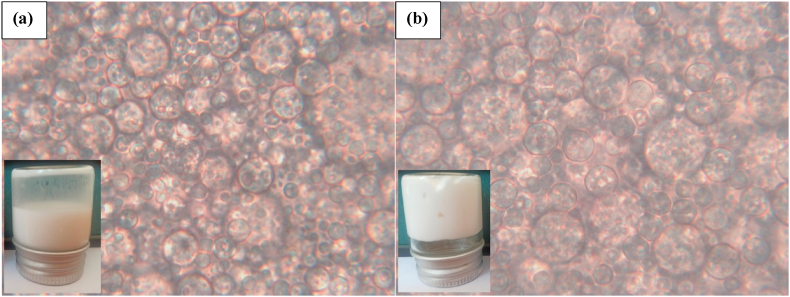
Micrographs and visual appearances of (a) water in oil in water double emulsion containing 0.75% salt in W_1_ and (b) water in gelled oil in water double emulsion containing 0.75% salt in W_1_.

Fig. 1 shows the microstructures of the DE3 and DE7 samples. Regardless of the physical state of the lipid phase, the successful formation of DEs in both samples was confirmed by detecting small aqueous droplets entrapped in the oil droplets. DE3 (Fig. 1a) had a relatively larger droplet size due to the higher possibility of oil and water droplets coalescence through the second emulsification process. In contrast, optical micrographs of DE7 showed smaller droplets, suggesting the reduction of the droplet coalescence after the gelation of the oil lipid. The good stability of DEs droplets against flocculation and coalescence during storage was also reported by Ghiasi et al. (2022) as affected by the both oil phase and inner aqueous phase gelation.

#### 3.1.2 Encapsulation efficiency of salt

According to Table 3, high encapsulation efficiency (>85%) of salt in the internal phase of DEs was determined for all samples. However, the gelation of the lipid phase of DEs (in DE6, DE7, and DE8) significantly improved salt encapsulation efficiency due to the rigidity and physical barrier properties of the oleogel structure which consequently slowed down salt release from the internal water droplets to the external ones after preparation and during storage. This protective effect of the oleogel network in the emulsion systems on the encapsulated ingredients was also previously reported for iron and cuminaldehyde (Ghiasi et al., 2019, 2022). In contrast, the higher fluidity of the liquid oil phase in DE2, DE3, and DE4 samples led to easier salt diffusion and leakage to the external water droplets, resulting in a lower encapsulation efficiency. Similarly, Ghiasi et al. (2021a) observed a reduction in encapsulation efficiency and loading capacity for encapsulated vitamin D_3_ inside the fluid bilayer shell of nanoliposomes compared to those stabilized by a gel network inside the lipidic bilayer.

**Table 3:** Encapsulation efficiency (EE) of different double emulsion samples.

Sample	EE (%)
**DE1**	–
**DE2**	86.45 ± 0.77*^E^
**DE3**	88.14 ± 0.51^D^
**DE4**	89.98 ± 0.34^C^
**DE5**	–
**DE6**	94.41 ± 1.01^B^
**DE7**	96.73 ± 1.45^A^
**DE8**	97.62 ± 0.71^A^

DE1:1% salt in external water phase/liquid oil phase; DE2: 1% salt in internal water phase/liquid oil phase; DE3: 0.75% salt in internal water phase/liquid oil phase; DE4: 0.5% salt in internal water phase/liquid oil phase; DE5: 1% salt in external water phase/gelled oil phase; DE6: 1% salt in internal water phase/gelled oil phase; DE7: 0.75% salt in internal water phase/gelled oil phase; DE8: 0.5% salt in internal water phase/gelled oil phase.

* Data represent mean ± standard deviation of three independent repeats. Different letters in each column indicate significant differences (*P* < 0.05).

#### 3.1.3 Viscosity

**Fig. 2:**
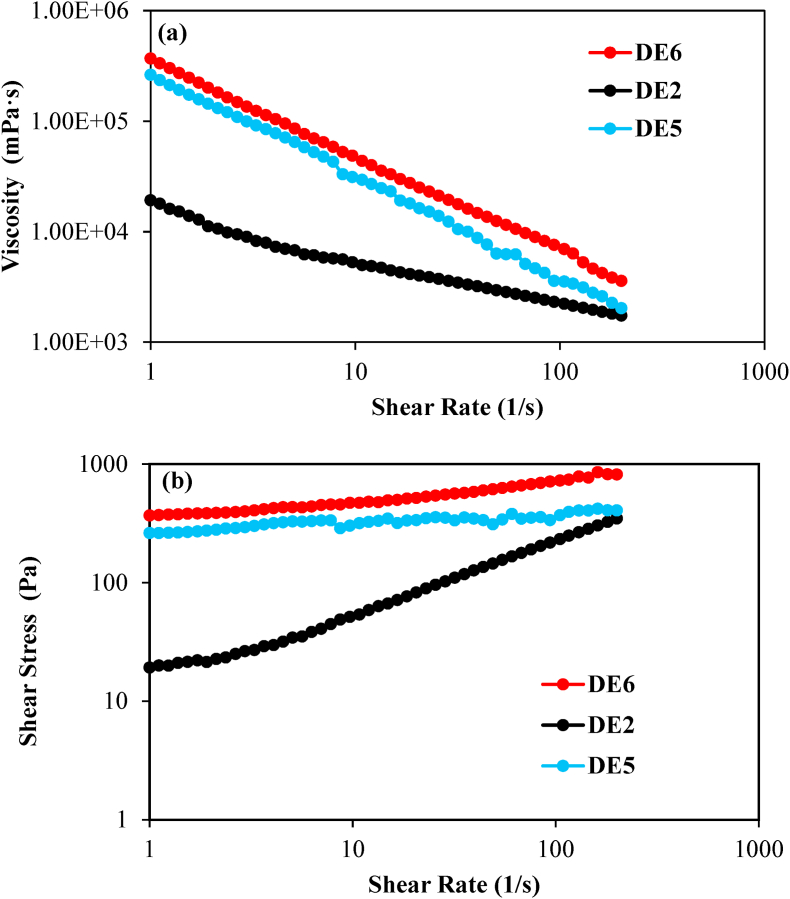
(a) Viscosity and (b) shear stress curve of water in oil in water double emulsion containing 1% salt in W_1_ (DE2), water in gelled oil in water double emulsion containing 1% salt in W_2_ (DE5), and water in gelled oil in water double emulsion containing 1% salt in W_1_ (DE6).

According to Fig. 2, a significant reduction of the apparent viscosity was observed by increasing the shear rate in all DE2, DE5, and DE6 samples, suggesting their strong shear thinning behavior. This characteristic was attributed to the destruction of the intermolecular interactions at high shear stress (Ghiasi and Golmakani, 2022b). According to Fig. 2, the apparent viscosity of DE5 and DE6 were significantly higher than DE2, indicating the remarkable effect of the lipid phase gelation on the final viscosity. Consequently, the formation of a compact and strong oleogel structure in oil droplets can lead to a greater resistance of the sample to flow in the direction of the probe rotation (Ghiasi et al., 2021b). The incorporation of salt, either in the W_1_ or W_2_, showed no significant result on the apparent viscosity (*P* ≥ 0.05) at low shear rates. However, by increasing the shear rate, higher viscosity values were observed for DE6 than DE5. This observation could be related to better DE stability by the existence of an appropriate concentration of salt in the W_1_ droplets. Indeed, the presence of salt in W_1_ plays a critical role in preventing water droplet coalescence in primary emulsions by counter balancing the Laplace pressure differences between droplets and also in balancing the osmotic pressure difference (Wankhede et al., 2020). As shown in Fig. 2, the increase in shear stress at higher shear rates for all samples could be related to more friction between parallel sliding plates, which was more significant for DE5 and DE6 samples with gelled lipid phase.

### 3.2 Burger properties

#### 3.2.1 pH

**Fig. 3:**
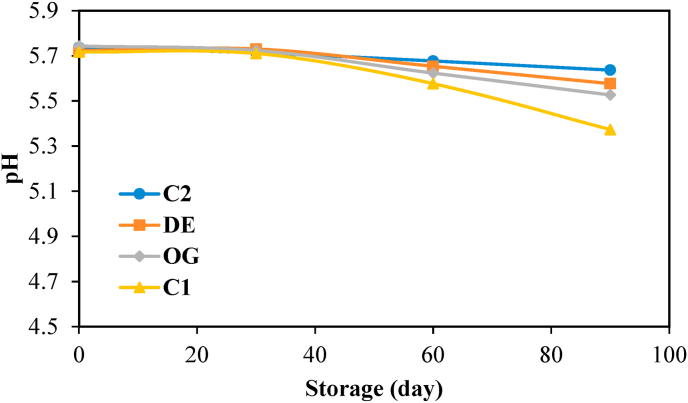
pH of control burger sample (C1), industrial control sample containing BHT (C2), and those formulated with oleogel (OG) and water in gelled oil in water double emulsion containing 0.75% salt in W_1_ (DE7).

According to Fig. 3, the pH values of all burgers remained constant during one month of storage. Also, the pH values of samples showed no significant differences for up to one month. After that, a gradual decrease in pH was observed in all burger samples up to the end of storage. However, this reduction was less pronounced for the sample containing synthetic antioxidant BHT, while the highest pH reduction was observed for the control sample due to the higher fat hydrolysis and free fatty acid formation during the storage period under the absence of antioxidant ingredients. On the other hand, the presence of cinnamaldehyde in those samples formulated with oleogel and DE7 improved oxidative stability. However, cinnamaldehyde exhibited lower scavenging activity as compared to synthetic BHT.

#### 3.2.2 Color

**Fig. 4:**
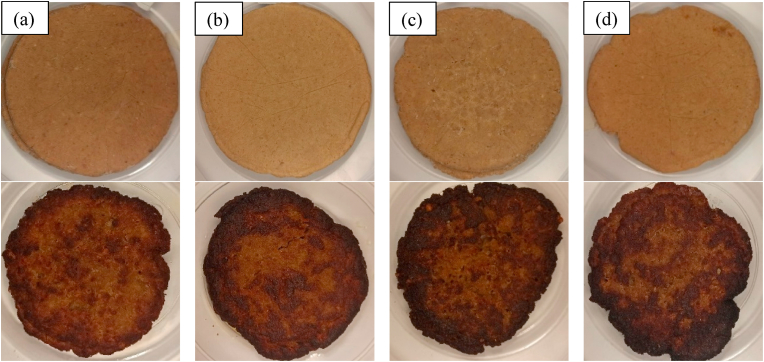
Appearance of raw and fried burger samples. (a) control, (b) formulated with water in gelled oil in water double emulsion containing 0.75% salt in W_1_ (DE7), (c) formulated with oleogel, and (d) industrial control sample containing BHT.

**Fig. 5:**
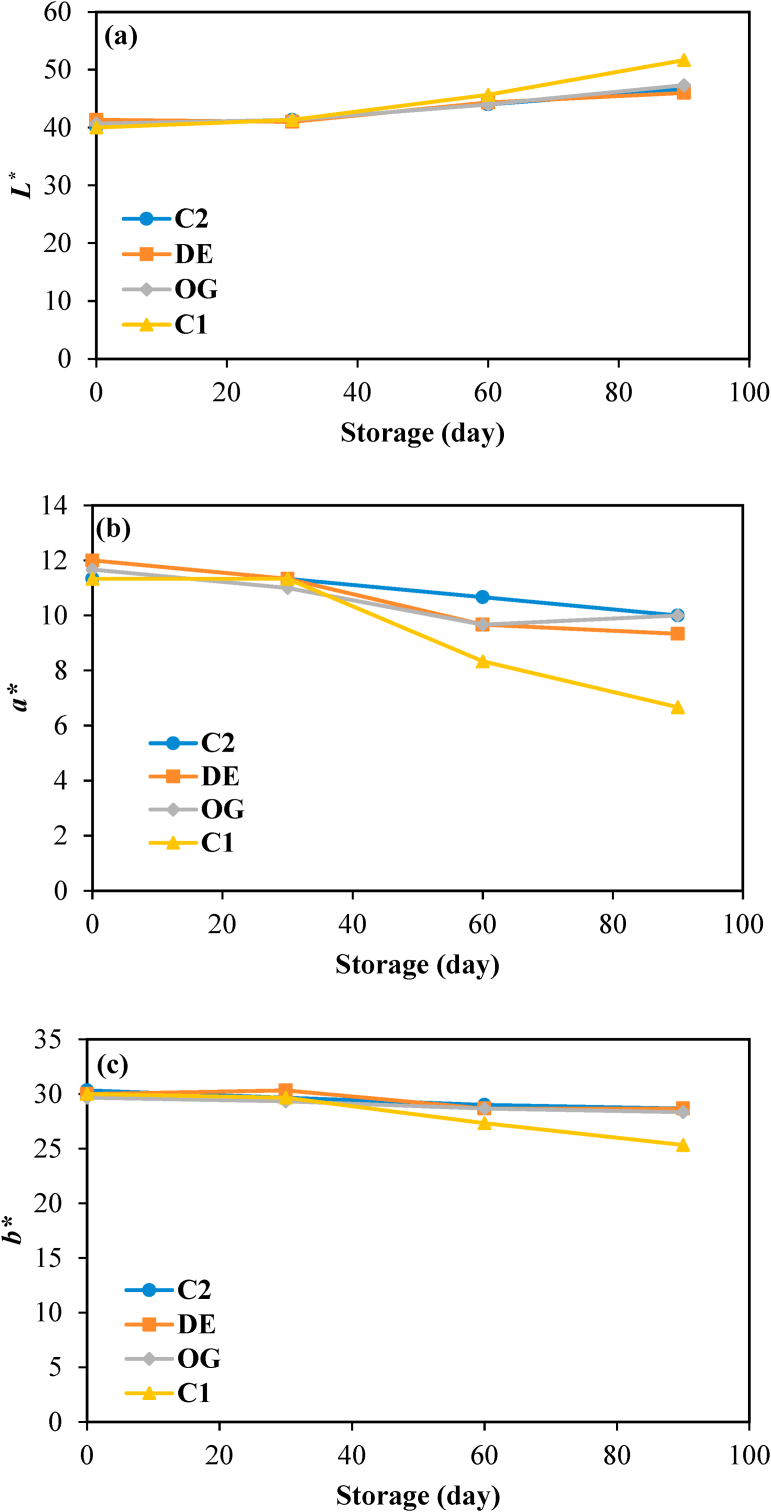
Color properties (L* (a), a* (b) and b* (c)) of raw control burger samples (C1), those formulated with oleogel (OG) and water in gelled oil in water double emulsion containing 0.75% salt in W_1_ (DE7), and industrial control sample containing BHT (C2) during storage. (For interpretation of the references to color in this figure legend, the reader is referred to the Web version of this article.)

**Fig. 6:**
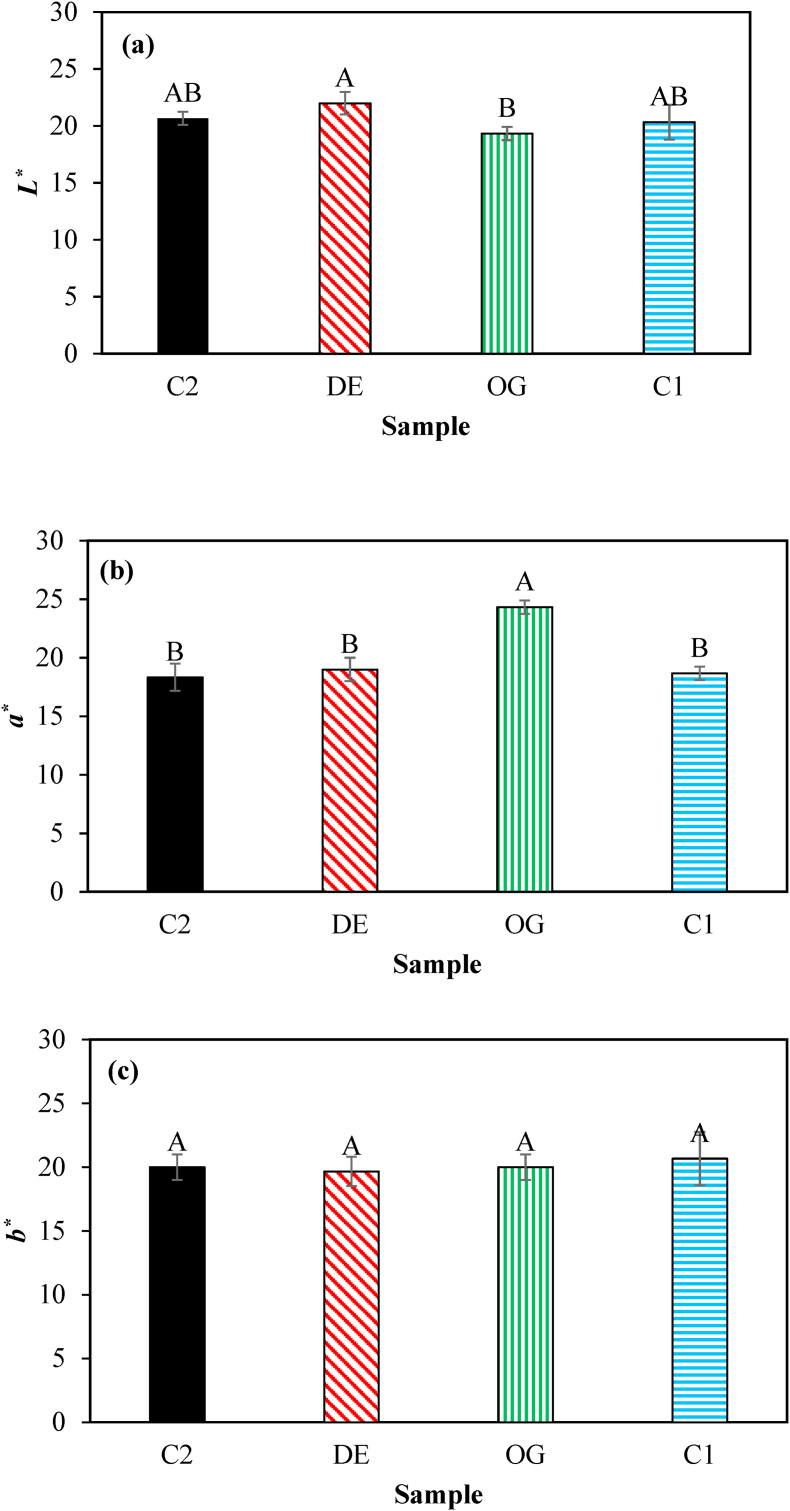
Color properties (L* (a), a* (b) and b* (c)) of fried control burger samples (C1), those formulated with oleogel (OG) and water in gelled oil in water double emulsion containing 0.75% salt in W_1_ (DE7), and industrial control sample containing BHT (C2). Different letters indicate significant differences (*P* < 0.05). (For interpretation of the references to color in this figure legend, the reader is referred to the Web version of this article.)

Color properties of burgers considerably affect consumer acceptance which is mainly induced by the presence of pigments as well as meat composition and texture. The visual appearances of raw and cooked burgers are shown in Fig. 4. According to Fig. 5, no significant changes in L*, a*, and b* values were evident during the first month of storage in all samples. After that, lightness increased while both redness and yellowness decreased up to the end of storage which was more significant for the control sample. This fact could be related to myoglobin oxidation and the production of brown metmyoglobin pigment as reported by Hashemi Gahruie et al. (2017). However, the presence of cinnamaldehyde and BHT in those burgers formulated with DE7 and oleogel limited the color changes of the raw samples during long-term frozen storage due to the oxidation delay of oxymyoglobin to metmyoglobin through binding transition metals involved in the production of free radicals and/or inhibiting free radicals (Barbieri et al., 2021). In the cooked burgers (Fig. 6), all samples had relatively similar values of color parameters (*P* ≥ 0.05) except for the sample formulated with oleogel in which relatively less lightness and more redness were observed. This is probably due to the improvement of heterogeneity in the texture, resulting in more light scattering inside the beef burgers and hence lower lightness. Moghtadaei et al. (2018) also reported similar effects after the partial substitution of animal fat in beef burgers with beeswax-based sesame oil oleogel. Franco et al. (2020) also reported an increase in redness in fermented sausages after the partial replacement of pork backfat with beeswax-based oleogel.

#### 3.2.3 Texture analysis

**Fig. 7:**
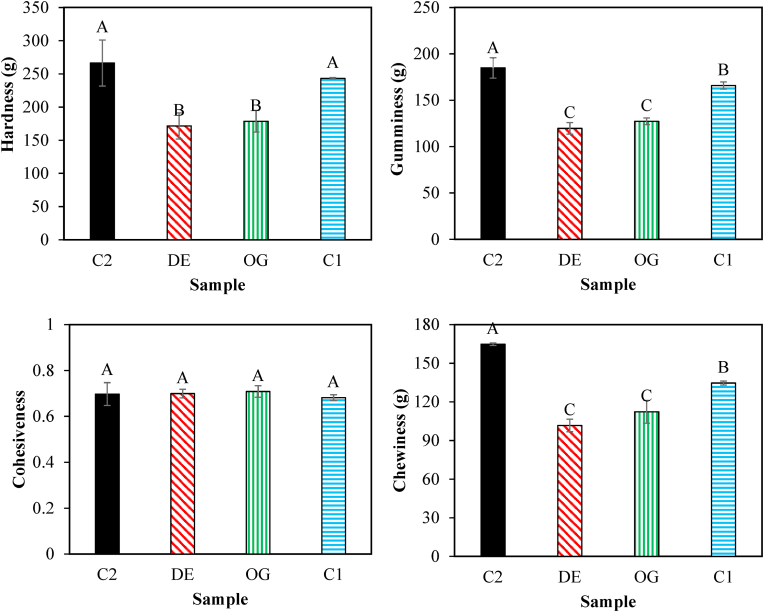
Texture properties of fried control burger samples (C1), those formulated with oleogel (OG) and water in gelled oil in water double emulsion containing 0.75% salt in W_1_ (DE7), and industrial control sample containing BHT (C2). Different letters indicate significant differences (*P* < 0.05).

According to Fig. 7, the hardness, chewiness, and gumminess of burgers were significantly affected by the partial replacement of animal fat with both oleogel and DE7. However, this replacement had no significant effects on cohesiveness (Fig. 7b). The presence of larger fat globules in oleogel fraction could be responsible for a significant reduction in mechanical parameters which was in good agreement with previous reports on low-fat pork burgers, beef burgers, and chicken patties formulated with oleogel or emulsions containing oleogel (Moghtadaei et al., 2018; Serdaroğlu et al., 2017). However, Tabibiazar et al. (2020) reported higher values of burger hardness and chewiness after 50% replacement of animal fat with carnauba wax/adipic acid oleogel. This conflict results could be related due to the application of different oleogel networks prepared with various gelators. On the other hand, the animal fat reduction in burger prepared with DE7 as well as the presence of water in the emulsion structure caused the weaker structure compared to the control burger (Özer and Çelegen, 2021).

#### 3.2.4 Cooking properties

**Fig. 8:**
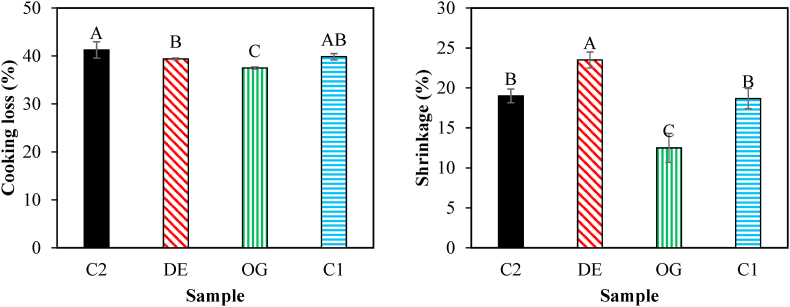
Cooking loss and shrinkage of control burger samples (C1), those formulated with oleogel (OG) and water in gelled oil in water double emulsion containing 0.75% salt in W_1_ (DE7), and industrial control sample containing BHT (C2). Different letters indicate significant differences (*P* < 0.05).

Cooking loss and cooking shrinkage of burgers were remarkably changed after incorporating oleogel and DE7 into the burgers formulations (Fig. 8). The highest cooking loss was observed in the control burger and it was decreased significantly in low animal fat burgers (Fig. 8a) due to the resistance of the oleogel structure against moisture loss. Indeed, the gel structure in those samples formulated with oleogel and DE7 acts like a physical barrier for water transferring through meat texture due to the formation of strong intermolecular interactions and high packing networks of fat globules (Ghiasi and Golmakani, 2022a). Similarly, Khalil (2000) also reported more water retention in low-fat burgers due to the formation of a denser protein matrix after fat reduction. According to Fig. 8b, BHT had no significant effect on cooking loss.

On the other hand, the control burger showed less cooking shrinkage, while the fat replacement by oleogel and DE7 led to a noticeable increment in cooking shrinkage (Fig. 8b). The fact can be related to the low melting point of the oleogel which is destroyed during frying, leading to higher oil leakage and consequently lower burger shrinkage. A similar trend was also observed by Moghtadaei et al. (2018) for 25% and 50% replacement of animal fat with beeswax oleogel in the beef burger.

#### 3.2.5 Oxidative stability

**Fig. 9:**
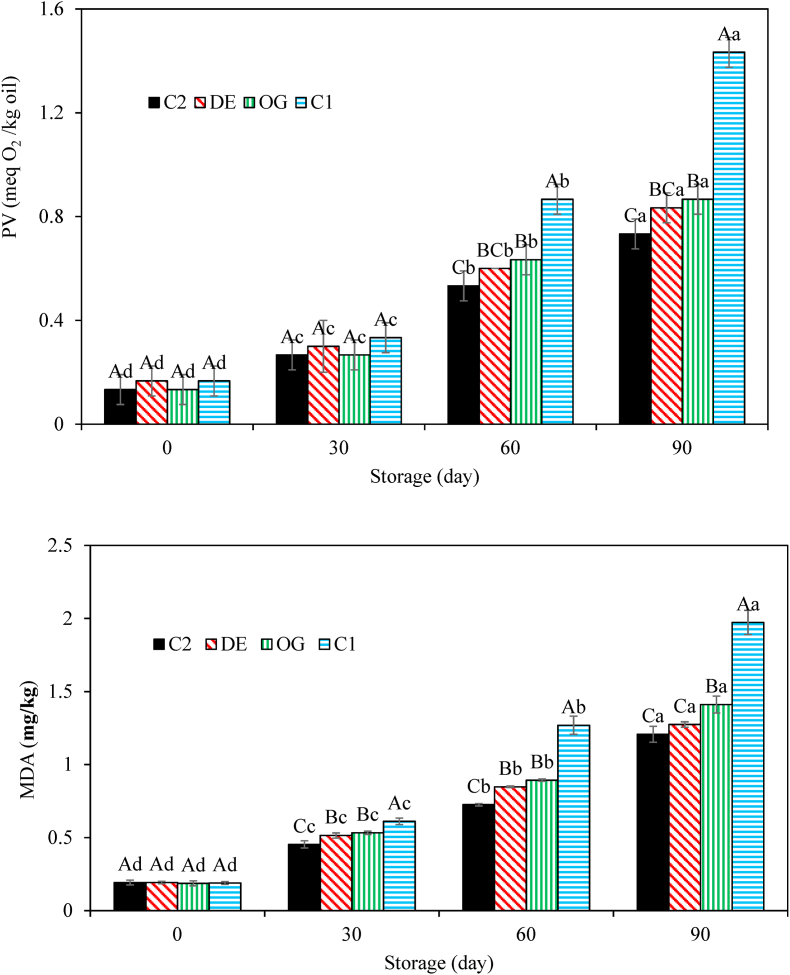
Peroxide and TBA values of raw control burger samples (C1), those formulated with oleogel (OG) and water in gelled oil in water double emulsion containing 0.75% salt in W_1_ (DE7), and industrial control sample containing BHT (C2) during storage. Different capital and small letters indicate significant differences (*P* < 0.05) between samples and during storage for each sample, respectively.

The results of primary oxidation (PV) and secondary oxidation (TBARS) are presented in Fig. 9a and b, respectively. During one month of frozen storage, the remarkable increases in both PV and TBARS values in all samples indicated the significant effect of time on oxidative stability. Additionally, the lowest oxidative stability was related to the animal fat burger and the partial replacement of oleogel and DE7 led to considerable reductions in PV and TBARS values due to the existence of cinnamaldehyde which slowed down the oxidation reaction. The addition of BHT into the animal fat burger also prompted oxidative stability which was more pronounced than cinnamaldehyde. Similar results have been reported regarding the stronger antioxidant capacity of synthetic antioxidants compared to natural ones (Ghiasi et al., 2021b; Ribeiro et al., 2019).

#### 3.2.6 Sensory evaluation

**Fig. 10:**
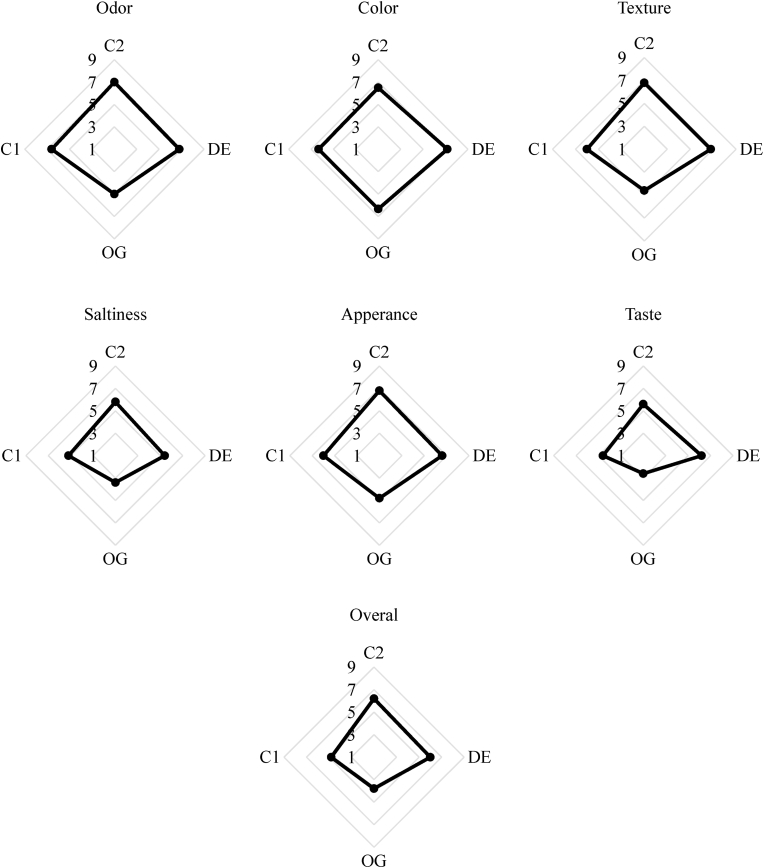
Sensory properties of control burger samples (C1), those formulated with oleogel (OG) and water in gelled oil in water double emulsion containing 0.75% salt in W_1_ (DE7), and industrial control sample containing BHT (C2).

As shown in Fig. 10, high color scores were obtained for all samples and no differences were found between the color of the control burger with other samples which was in good agreement with a previous report by Özer and Çelegen, 2021 for the replacement of olive oil oleogel emulsion in burgers. However, the partial replacement of animal fat with oleogel led to negative effects on the texture due to its weaker texture compared to the control sample. In contrast, the control burger with animal fat and those formulated with BHT (as the commercial burger) and DE7 had high scores for texture. In addition, low taste and flavor scores were obtained for burger samples containing oleogel, as panelists could recognize a lipid oxidation off-flavor which could be related to the higher levels of unsaturated fat formed during oil heating. However, this can be minimized by reformulating spices. In addition, no significant differences in flavor of the animal fat burger incorporated with BHT and those formulated with DE7 were reported. Panelists preferred the flavor of animal fat burgers incorporated with BHT in comparison with the control sample. This preference might be due to the highest oxidation stability of burgers in the presence of BHT during storage which limits the formation of oxidized taste and aroma. In relation to the level of saltiness perception, acceptable scores were found for the control animal fat burger and those incorporated with BHT and DE7. Therefore, the encapsulation of salt in W_1_ droplets of DE stabilized with oleogel successfully presented a saltiness-enhancing effect on low-saturated fat and low-salt beef burgers. Therefore, salt is released in a desired and controlled profile during chewing, leading to an increased residence time of salt in the mouth and saltiness perception. In contrast, the healthier burger formulated with oleogel was significantly different in saltiness, as lower perceived saltiness was reported by the panelists. Regarding the overall acceptability, the highest scores correspond to the animal fat burger incorporated with BHT and DE7 followed by the control sample and burger formulated with oleogel. Therefore, it can be concluded that the taste contrast approach using double emulsion stabilized with oleogel can successfully reduce both the total saturated fat and sodium chloride contents in the formulations of beef burgers without affecting structural and sensory quality, particularly the saltiness.

## 4 Conclusion

In this research, W_1_/O/W_2_ double emulsions stabilized by a gel structure in the lipid phase were successfully used to encapsulate salt to improve the salty taste in low-sodium burgers as well as animal fat reduction. For this purpose, the effects of the oil phase gelation and the presence of different percentages of salt in the internal aqueous phase were first investigated on the stability, encapsulation capacity, and physicochemical characteristics of the double emulsions and then compared with the sample containing salt in the external aqueous phase. The results showed that the development of an oleogel structure in the oil phase significantly protected the salt against leakage; however, the higher fluidity of the oil phase in the convectional DEs led to salt leakage and less encapsulation efficiency. Regarding the flow behavior, all samples showed shear-thinning characteristics and the gelation of the oil phase had a more significant effect on the viscosity of DEs than the location of salt. Optical micrographs confirmed the successful preparation of DEs with a lower size distribution in those stabilized with an oleogel network. The preparation of low-sodium burgers with the best formulation of DEs incorporating cinnamaldehyde was then performed to compare the physical and sensory properties against the control animal fat burger and the burger sample formulated with cinnamaldehyde-loaded oleogel as a partial replacement of animal fat. The presence of cinnamaldehyde resulted in significantly higher oxidative stability of low-saturated fat burgers. Moreover, the oxidation of myoglobin to brown metmyoglobin led to significant color changes in the control animal fat burger, while the color parameters of healthier burgers remained relatively constant during long-term frozen storage. The partial replacements of the oleogel and DE in beef burgers reduced cooking loss as a positive aspect. However, low-animal fat samples could not mimic the textural properties of the control burger. The sensory analysis showed that panelists preferred burgers formulated with DE compared to animal fat burgers in terms of overall acceptability. In conclusion, the taste contrast approach using water in gelled oil in water double emulsion can be an efficient approach for modulating salt perception and producing low-sodium and low-fat foods while maintaining the saltiness intensity and consumer liking.

## CRediT authorship contribution statement

**Hadi Hashemi:** Conceptualization, Methodology, Software, Validation, Formal analysis, Data curation, Resources, Investigation, Writing – original draft, Writing – review & editing, Visualization, Project administration. **Mohammad Hadi Eskandari:** Conceptualization, Validation, Data curation, Resources, Writing – review & editing, Writing – original draft, Visualization, Project administration, Funding acquisition, Supervision. **Seyed Mohammad Hashem Hosseini:** Conceptualization, Validation, Data curation, Resources, Writing – review & editing, Writing – original draft, Visualization, Project administration, Funding acquisition, Supervision.

## Declaration of competing interest

The authors declare that they have no known competing financial interests or personal relationships that could have appeared to influence the work reported in this paper.

## Data Availability

Data will be made available on request.
